# Effects of hydrolyzed yeast on weanling pig growth performance, fecal dry matter, and stress-related blood antioxidant criteria

**DOI:** 10.1093/jas/skaf331

**Published:** 2025-09-26

**Authors:** Jessica L Smallfield, Joel M DeRouchey, Mike D Tokach, Jason C Woodworth, Robert D Goodband, Katelyn N Gaffield, Jordan T Gebhardt, Robin Yao, Yitong Guo

**Affiliations:** Department of Animal Sciences and Industry, College of Agriculture, Kansas State University, Manhattan, KS 66506-0201; Department of Animal Sciences and Industry, College of Agriculture, Kansas State University, Manhattan, KS 66506-0201; Department of Animal Sciences and Industry, College of Agriculture, Kansas State University, Manhattan, KS 66506-0201; Department of Animal Sciences and Industry, College of Agriculture, Kansas State University, Manhattan, KS 66506-0201; Department of Animal Sciences and Industry, College of Agriculture, Kansas State University, Manhattan, KS 66506-0201; Department of Animal Sciences and Industry, College of Agriculture, Kansas State University, Manhattan, KS 66506-0201; Department of Diagnostic Medicine/Pathobiology, College of Veterinary Medicine, Kansas State University, Manhattan, KS 66506-0201; Planet Bioscience, Boon Lay Way, Singapore; Sinagri YingTai Bio-peptide Co., Ltd, Linzhou City, China

**Keywords:** cytokine production, fecal dry matter, hydrolyzed yeast, nursery pig, superoxide dismutase, total antioxidant capacity

## Abstract

A total of 360 weanling pigs (241 × 600, DNA; initially 5.4 ± 0.01 kg) were used to evaluate the effects of a hydrolyzed yeast product (HY) on growth performance, fecal dry matter (DM), and stress-relevant blood antioxidant criteria. Pens of pigs were randomly allotted to one of six dietary treatments in a generalized randomized block design with 5 pigs per pen and 12 pens per treatment. Pigs were blocked with 4 replications of light (4.3 ± 0.01 kg), medium (5.4 ± 0.01 kg), and heavy (6.5 ± 0.01 kg) weight pens per treatment. Diets were corn-soybean meal-based and consisted of a negative control (NC) diet, a positive control (PC) diet which was the NC diet + 55 mg/kg carbadox, the PC diet with 0.04% HY, and the NC diet with either 0.04%, 0.08%, or 0.12% HY. Linear and quadratic contrasts were tested within increasing levels of HY in diets without carbadox. The main effect of carbadox was evaluated by comparing the average of 0% and 0.04% HY in the presence and absence of carbadox. Treatment diets were fed in three phases from day 0 to 10 (phase 1), day 10 to 24 (phase 2), and day 24 to 45 (phase 3). On day 10 and 24, fecal samples were collected from the same three initially randomly selected pigs in each pen to determine fecal DM and fecal scores. Blood samples were collected on day 10 and 45 from the same representative pig in each pen for total antioxidant capacity (TAC) and superoxide dismutase (SOD). Overall (day 0 to 45), final weight increased (*P *< 0.05) while average daily gain (ADG) and average daily feed intake (ADFI) tended to increase (*P *< 0.10) for pigs fed diets containing carbadox compared to pigs fed diets without carbadox. Gain:feed ratio (G:F) increased (*P *= 0.017) when 0.04% HY was added to the negative and positive control diets. On day 10, increasing HY in diets without carbadox increased fecal DM (linear, *P *= 0.005). Additionally, pigs fed carbadox had increased (*P *< 0.05) fecal DM on day 10 and 24 compared to pigs not fed carbadox. There were no treatment differences observed on day 10 for TAC; however, TAC decreased (linear, *P *= 0.008) on day 45 as HY increased in the diet. Increasing HY tended to decrease (linear, *P *= 0.095) serum SOD activity on day 10 while no treatment differences were observed on day 45. In summary, pigs fed carbadox had increased overall ADG and fecal DM. Increasing HY inclusion did not affect growth performance; however, fecal DM was improved during the early nursery period. Additionally, feeding 0.04% HY improved overall G:F.

## Teaser

The *Saccharomyces cerevisiae* yeast-based product evaluated improved feed efficiency and affected cytokine production by reducing pro-inflammatory serum cytokine concentrations.

## Introduction

Weaned pigs often experience reduced growth performance and increased prevalence of diarrhea due to the transition from a milk-based diet to a dry, cereal-based diet (Kluess et al., 2010; Campbell et al., 2013). At weaning, beneficial *Lactobacillus* populations are reduced, which compromises gut barrier integrity and allows pathogenic bacteria, like *Escherichia coli*, to proliferate (Fairbrother et al., 2005). The proliferation of the *E. coli* pathotype, enterotoxigenic *E. coli* (ETEC), is a significant contributor to postweaning diarrhea which can adversely affect intestinal integrity and growth performance (Yan et al., 2024). To help overcome these challenges and maintain weanling pig health, in-feed antibiotics have been widely utilized to maintain gut microbiota and improve growth performance.

Carbadox, a quinoxaline class of antimicrobials, is used to control and prevent enteric health challenges and improve growth performance in pigs (Dasenaki et al., 2023). It inhibits bacterial growth by intercalating into DNA, leading to mutations that hinder replication (Chen et al., 2009). At low doses, carbadox can be used to improve feed efficiency (Dritz et al., 2002). Due to concerns of antibiotic resistance, the U.S. Food and Drug Administration prohibited the use of certain in-feed antibiotics important for human medicine as growth promoters in livestock in 2017 (FDA; Food and Drug Administration), 2013). This ban on antibiotic growth promoters (AGP) has driven interest in finding alternatives with similar health benefits.

Nutritional strategies that may act as a substitute for AGP such as probiotics, prebiotics, phytogenic additives, and organic acids have been extensively explored. Yeast-based feed additives, specifically *Saccharomyces cerevisiae*, have been of interest due to their potential to improve gut health, nutrient digestibility, and growth performance (Schiavone et al., 2015; Sampath et al., 2023). There are different kinds of yeast products such as yeast extracts, yeast cultures, yeast cell walls, yeast cell wall components, and live yeast cells. Previous literature has shown improvements in intestinal morphology, diarrhea prevalence, and intestinal inflammation when utilizing dietary yeast products during the nursery phase (Shen et al., 2009; Zanello et al., 2011; Trckova et al., 2014). However, studies measuring growth performance have been variable. Van Der Peet-Schwering et al. (2007) and Shen et al. (2009) observed improvements in growth performance when yeast culture was utilized in the diet, while others using live yeast have shown no benefit (van Heugten et al., 2003; Chance et al., 2021). These inconsistent findings could stem from variations in the types of yeast products used, the inclusion and duration of supplementation, diet composition, and health status of the pigs.

A novel hydrolyzed yeast product (HY; Ceretide S, Boon Lay Way, Singapore) is derived from cultivated *S. cerevisiae* yeast cells, which undergo enzymatic hydrolysis using exogenous enzymes. This process results in a product that contains the whole-cell components of yeast resulting in a composition that also contains metabolic by-products such as enzymes, oligopeptides, vitamins, saccharides, organic acids, and other fermentation metabolites. Hydrolyzed yeast is thought to combine the beneficial aspects of yeast extract including various soluble nutrients and yeast cell walls containing mannan-oligosaccharides (MOS) and β-glucans. Because these components in HY act as immunomodulators, they can alter cytokine production reducing pro-inflammatory responses (Zanello et al., 2011). This may enhance gut health and nutrition utilization, leading to improvements in growth performance (Boontiam et al., 2022).

Given the wide variety of yeast products available, it is important to evaluate the efficacy of new products. Therefore, the objective of this study was to evaluate the effects of HY with or without carbadox on growth performance, fecal dry matter (DM), stress-related blood antioxidant criteria, and serum cytokine concentrations in nursery pigs. It was hypothesized that the addition of HY would improve nursery pig growth performance and fecal DM.

## Materials and Methods

The Kansas State University Institutional Animal Care and Use Committee approved the protocols used in this experiment (IACUC #4942).

This study was conducted at the Kansas State University Swine Research and Teaching Center in Manhattan, KS. A total of 360 weanling pigs (241 × 600 DNA; initially 5.4 ± 0.01 kg) were used in a 45-d growth trial. Pigs were weaned at approximately 18 d of age and blocked by initial body weight **(BW)**. Pens of pigs were randomly allotted to one of six dietary treatments in a generalized randomized block design. There were five pigs per pen, and within each block, there were four pens per weight group [light (4.3 ± 0.01 kg), medium (5.4 ± 0.01 kg), and heavy (6.5 ± 0.01 kg)] for a total of 12 replications per treatment. Diets were corn-soybean meal-based and consisted of a negative control (NC) diet, a positive control (PC) diet which was the NC diet + 55 mg/kg carbadox (Mecadox 2.5, Phibro; Teaneck, NJ), the PC diet with 0.04% HY, and the NC diet with either 0.04%, 0.08%, or 0.12% HY (Table 1). All diets were formulated to 1.35% standardized ileal digestible (SID) Lys in phases 1 and 2 and 1.30% SID Lys in phase 3. All diets met or exceeded the nutrient requirement estimates of other amino acids as a ratio to Lys (NRC, 2012). Pigs were fed treatment diets in three phases from day 0 to 10 (phase 1), day 10 to 24 (phase 2), and day 24 to 45 (phase 3). Treatment diets were manufactured at the Kansas State University O.H. Kruse Feed Technology Innovation Center in Manhattan, KS and fed in meal form for all phases. Each pen contained a 3-hole, dry self-feeder and a nipple waterer for *ad libitum* access to feed and water.

**Table 1.** Composition of experimental diets (as-fed basis)^1^

	Phase 1		Phase 2		Phase 3	
Ingredient, % Carbadox:	Yes	No	Yes	No	Yes	No
**Corn**	45.45	46.34	56.95	57.84	65.34	66.24
** Soybean meal (47% CP)**	16.13	16.07	23.92	23.86	29.81	29.75
** Enzymatically treated SBM^2^**	5.00	5.00	4.50	4.50	---	---
**Whey powder**	20.00	20.00	10.00	10.00	---	---
**Fish meal**	4.50	4.50	---	---	---	---
**Lactose**	4.50	4.50	---	---	---	---
**Soybean oil**	1.00	1.00	---	---	---	---
**Calcium carbonate**	0.20	0.37	0.53	0.70	0.68	0.85
**Monocalcium P (21% P)**	0.45	0.45	1.00	1.00	1.05	1.05
**Salt**	0.33	0.33	0.55	0.55	0.60	0.60
**L-Lys-HCl, 78.8%**	0.43	0.43	0.50	0.50	0.50	0.50
**DL-Met, 98.5%**	0.22	0.22	0.23	0.23	0.20	0.20
**L-Thr, 98.5%**	0.21	0.21	0.23	0.23	0.23	0.23
**L-Trp, 98.0%**	0.04	0.04	0.03	0.03	0.03	0.03
**L-Val, 96.5 %**	0.14	0.14	0.14	0.14	0.14	0.14
**Trace mineral premix**	0.15	0.15	0.15	0.15	0.15	0.15
** Vitamin premix**	0.25	0.25	0.25	0.25	0.25	0.25
** Phytase^3^**	0.03	0.03	0.03	0.03	0.03	0.03
** Carbadox^4^**	1.00	---	1.00	---	1.00	---
** Hydrolyzed yeast^5^**	±	±	±	±	±	±
**Total**	100	100	100	100	100	100
**Calculated analysis**						
** SID Lys, %^6^**	1.35	1.35	1.35	1.35	1.30	1.30
** Net energy, kcal/kg**	2,555	2,576	2,434	2,455	2,407	2,428
** Crude protein, %**	20.2	20.2	20.7	20.7	20.5	20.5
** Ca, %**	0.71	0.71	0.71	0.71	0.72	0.72
** STTD P, %^7^**	0.44	0.44	0.41	0.41	0.36	0.36
** Ca:P**	1.11	1.11	1.11	1.11	1.18	1.18

1 Phase 1 diets were fed from day 0 to 10 (5.4 to 6.2 kg). Phase 2 diets were fed from day 10 to 24 (6.2 to 11.6 kg). Phase 3 diets were fed from day 24 to 45 (11.6 to 26.8 kg).

2 ESBM; enzymatically treated soybean meal; HP 300 (Hamlet Protein, Findlay, OH).

3 Ronozyme HiPhos 2700 (DSM, Parsippany, NJ) added at 811 FTU/kg provided an estimated release of 0.14% STTD P.

4 Mecadox 2.5 (Phibro; Teaneck, NJ) included at 55 mg/kg of complete feed.

5 Ceretide S (Planet Bioscience; Boon Lay Way, Singapore) added at the expense of corn.

6 Standardized ileal digestible.

7 Standardized total tract digestible.

Pig weights and feed disappearance were measured on day 0, 10, 24, and 45 to determine average daily gain (ADG), average daily feed intake (ADFI), and gain:feed ratio (G:F). On day 10 and 24, fecal samples were collected from the same three initially randomly selected pigs in each pen to determine fecal DM and fecal score. Fecal samples were dried at 55°C in a forced air oven for 48 h, and the ratio of dried to wet fecal weight determined the percentage fecal DM. The average of the three samples from each pen was then used for statistical analysis. Fecal samples were also scored in the bag by three observers on day 10 and 24 using a 0 to 4 scoring system: 0 = hard, pellet-like lumps; 1 = firm, formed feces; 2 = normal feces; 3 = mild looseness; and 4 = diarrhea.

Blood samples were collected on day 10 and 45 from the same median-weight pig in each pen for total antioxidant capacity (TAC), superoxide dismutase (SOD), and serum cytokine concentrations. Blood samples were collected with blood collection tubes (Covidien Monoject blood collection tubes, silicone-coated tubes with red stoppers, no additive, 10 mL; Medtronic, Minneapolis, MN). The clotted samples were centrifuged at 1,500 × *g* for 30 min, after which the resulting serum supernatants were divided into 4 polypropylene tubes (PR1MA microcentrifuge tubes, natural boil-proof; Midwest Scientific, St Louis, MO) and stored as aliquots at −80°C until use. Serum cytokine panel analyses (GM-CSF, IFNγ, IL-1α, IL-1ra, IL-1β, IL-2, IL-4, IL-6, IL-8, IL-10, IL-12, IL-18, and TNFα) were conducted at Eve Technologies (Calgary, AB, Canada). Serum TAC and SOD analyses were conducted at the Kansas State University Swine Nutrition Laboratory (Manhattan, KS). Both serum SOD and TAC samples were analyzed in duplicate in 96-well microplates with an intra-assay CV ≤ 5.0%. Assay kits for SOD and TAC were from Cayman Chemical Company (Ann Arbor, MI; # 706002) and Cell Biolabs Inc. (San Diego, CA; # STA-360), respectively. Superoxide dismutase activity is assessed by measuring the dismutation of superoxide radicals generated by xanthine oxidase and hypoxanthine. Total antioxidant capacity is based on the reduction of copper(II) and copper(I). Both assays were read on a BioTek Epoch2 microplate spectrophotometer.

### Statistical analysis

Growth performance, fecal DM, and cytokine panel data were analyzed as a generalized randomized block design as a one-way ANOVA using the lmer function from the lme4 package in R studio (Version 4.3.1, R Core Team. Vienna, Austria) with pen serving as the experimental unit and dietary treatment and weight block as fixed effects. Linear and quadratic contrasts were tested within increasing levels of HY in diets without carbadox. Additionally, the main effect of HY was evaluated plus the interaction of HY and carbadox. The main effect of carbadox was evaluated by comparing the average of 0% and 0.04% HY in the presence and absence of carbadox. Additionally, a comparison was made between carbadox with 0.04% HY and carbadox without HY. Fecal DM and serum samples were analyzed using the fixed effects of day, treatment, block, and the associated interactions accounting for repeated measures over time.

For serum cytokine data, the data were analyzed with the raw fluorescence intensity value based on Breen et al. (2015) with a log10 transformation for statistical analysis, which was transformed back for reporting of treatment means. For serum TAC and SOD assays, data were analyzed using the GLIMMIX procedure of SAS OnDemand for Academics (SAS Institute Inc., Cary, NC) with microtiter plate used in the model as a random intercept. Fecal scores were summarized using the FREQ procedure of SAS OnDemand for Academics and reported as a percentage of observations within each score category by treatment. Fecal scores are reported using descriptive statistics due to a lack of model fit. When treatment was a significant source of variation, differences were determined by pairwise comparison using the Tukey-Kramer multiplicity adjustment to control for Type I error. Results were considered significant with *P *≤ 0.05 and marginally significant with 0.05 < *P *≤ 0.10.

## Results

There were no BW block interactions for growth performance, fecal DM, or serum antioxidant data, therefore, interpretation is based on the main effect of dietary treatment. There were no carbadox by HY interactions observed.

### Growth performance

For growth performance criteria, there were no linear or quadratic effects of increasing HY observed. From day 0 to 10 (phase 1), there was a main effect of carbadox, where pigs fed diets containing carbadox had increased (*P *≤ 0.025) ADG and day 10 BW compared to those not fed carbadox with either none or 0.04% HY (Table 2). From day 10 to 24 (phase 2), an increase in G:F was observed (*P *= 0.041) when 0.04% HY was added, regardless of carbadox inclusion. Also, day 24 BW was greater (*P *= 0.048) in pigs fed diets containing carbadox compared to pigs fed diets without carbadox. From day 24 to 45 (phase 3), ADFI increased (*P *= 0.041) for pigs fed diets containing carbadox compared to pigs not fed carbadox, which led to a tendency (*P *= 0.087) for increased ADG due to carbadox. Also, pigs fed 0.04% HY and carbadox tended to increase (*P *= 0.072) G:F compared to pigs fed carbadox without HY.

**Table 2.** Effects of hydrolyzed yeast (HY) and carbadox on growth performance and fecal dry matter (DM) of nursery pigs^1^

								*P^2^*			
Carbadox^3^:	Yes		No					HY in No Carbadox^4^			
HY, %:	0.00	0.04	0.00	0.04	0.08	0.12	SEM	Linear	Quadratic	0.04% HY^5^	Carbadox^6^
Body weight, kg											
day 0	5.4	5.4	5.4	5.4	5.4	5.4	0.01	0.224	0.379	0.282	0.232
day 10	6.3	6.4	6.1	6.0	6.1	6.0	0.11	0.878	0.880	0.905	0.018
day 24	11.9	12.3	11.5	11.4	11.2	11.4	0.32	0.657	0.616	0.749	0.048
day 45	27.2	28.2	26.5	26.5	26.0	26.1	0.50	0.388	0.909	0.375	0.026
Phase 1 (day 0 to 10)											
ADG, g^7^	92	96	71	67	71	65	11.4	0.773	0.958	0.999	0.025
ADFI, g^8^	132	121	122	113	112	105	9.8	0.234	0.901	0.295	0.353
G:F, g/kg^9^	676	687	573	552	503	582	99.6	0.959	0.604	0.960	0.223
Phase 2 (day 10 to 24)											
ADG, g	399	408	375	380	350	371	18.3	0.618	0.652	0.697	0.141
ADFI, g	543	546	522	507	494	511	24.0	0.660	0.492	0.790	0.204
G:F, g/kg	732	750	716	754	710	724	14.2	0.772	0.389	0.041	0.654
Phase 3 (day 24 to 45)											
ADG, g	728	751	714	721	703	699	12.9	0.269	0.686	0.248	0.087
ADFI, g	1,072	1,073	1,022	1,028	1,012	1,004	23.6	0.502	0.734	0.877	0.041
G:F, g/kg^10^	680	702	699	705	698	699	8.9	0.850	0.827	0.122	0.213
Overall (day 0 to 45)											
ADG, g	482	490	458	469	444	451	12.0	0.359	0.854	0.430	0.058
ADFI, g	696	685	656	663	639	643	18.7	0.435	0.932	0.906	0.089
G:F, g/kg^11^	694	717	699	711	697	703	7.7	0.937	0.645	0.017	0.943
Fecal DM, %^12^											
day 10	17.6	17.3	12.3	15.0	16.3	17.3	1.29	0.005	0.493	0.335	0.003
day 24	20.6	18.8	16.2	16.7	18.3	18.2	1.29	0.177	0.831	0.578	0.009

1 A total of 360 pigs (initially 5.4 ± 0.01 kg) were used in a 45-d growth study with 5 pigs per pen and 12 replicates per treatment (*N* = 12).

2 No interactive responses (*P *> 0.10) of carbadox and HY were observed.

3 Mecadox 2.5 (Phibro; Teaneck, NJ) included at 55 mg/kg of complete feed.

4 Increasing HY in diets without carbadox.

5 Comparing the mean of 0% HY with and without carbadox vs. the mean of 0.04% HY with and without carbadox.

6 Comparing the mean of 0% and 0.04% HY with carbadox vs. the mean of 0% and 0.04% HY without carbadox.

7 Average daily gain.

8 Average daily feed intake.

9 Gain:feed ratio.

10,11 Pairwise comparison of carbadox with 0.04% HY vs. carbadox without HY, ^10^*P* < 0.10. ^11^*P* < 0.05.

12 No treatment × day interactions (*P *> 0.10).

Overall (day 0 to 45), final BW increased (*P *= 0.026) and ADG and ADFI tended to increase (*P *≤ 0.089) for pigs fed diets containing carbadox compared to pigs fed diets without carbadox with either none or 0.04% HY. Gain-to-feed ratio increased (*P *= 0.017) when 0.04% HY was added, regardless of carbadox inclusion. The combination of 0.04% HY and carbadox increased (*P *< 0.05) G:F compared to pigs fed carbadox alone.

### Fecal DM and scoring

**Figure 1.**
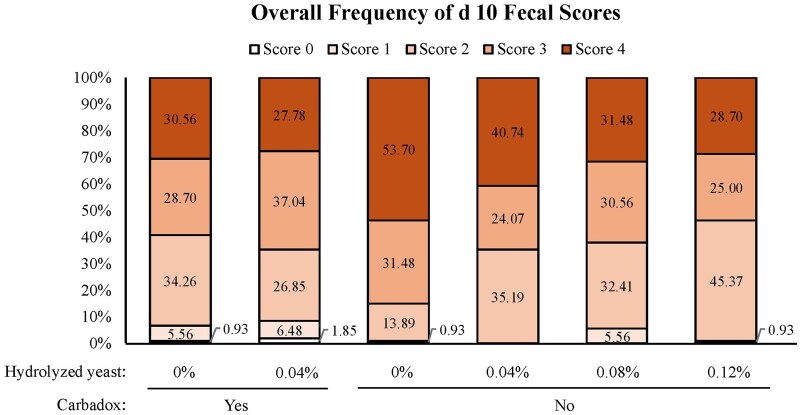
Day 10 overall frequency of fecal scores. Fecal scores are presented on a 4-point scale: 0 = hard, pellet-like lumps; 1 = firm, formed feces; 2 = normal feces; 3 = mild looseness; and 4 = diarrhea as the mean determined by three observers. Fecal scores are reported using descriptive statistics due to a lack of model fit.

**Figure 2.**
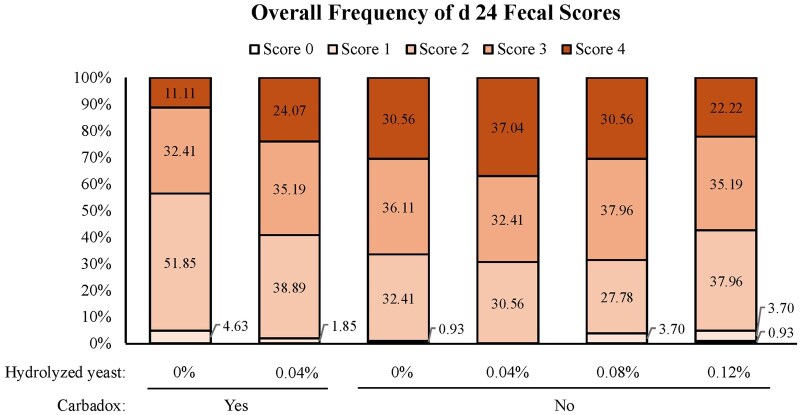
Day 24 overall frequency of fecal scores. Fecal scores are presented on a 4-point scale: 0 = hard, pellet-like lumps; 1 = firm, formed feces; 2 = normal feces; 3 = mild looseness; and 4 = diarrhea as the mean determined by three observers. Fecal scores are reported using descriptive statistics due to a lack of model fit.

On day 10, increasing HY in diets without carbadox increased (linear, *P *= 0.005) fecal DM. Additionally, pigs fed carbadox had increased (*P*  ≤ 0.009) fecal DM on day 10 and 24 compared to pigs not fed carbadox. Fecal scores are reported using descriptive statistics due to a lack of model fit. For fecal scoring on day 10, the pigs fed the negative control had a higher numerical incidence of diarrhea than pigs fed the other dietary treatments (Figure 1). The frequency of diarrhea (score of 4) decreased as HY increased in pigs fed diets either with or without carbadox. On day 24, pigs fed 0.04% HY numerically had the highest frequency of diarrhea with the frequency decreasing as the level of HY increased (Figure 2). Pigs fed the diet with carbadox alone had the lowest frequency of diarrhea.

### Serum cytokines

On day 10, pigs fed 0.04% HY in diets containing carbadox tended to have decreased (*P *< 0.10) cytokines IFNγ, IL-1α, IL-2, IL-4, IL-6, IL-10, and IL-18 compared to pigs fed carbadox alone (Table 3). Cytokines GM-CSF, IL-2, IL-4, and TNFα decreased then increased (quadratic, *P *< 0.05) on day 10 and IL-1α, IL-1β, IL-6, and IL-10 tended to decrease then increase (quadratic, *P *< 0.10) as HY increased in diets without carbadox with the lowest concentration observed at an inclusion level of 0.08% HY or 0.04% for GM-CSF. IFNγ increased (linear, *P *= 0.015) as HY increased on day 10 although a tendency for a quadratic response (quadratic, *P *= 0.065) was also observed. Cytokines IL-8 and IL-10 tended to decrease (*P *≤ 0.097) when 0.04% HY was added to the diet, regardless of carbadox inclusion. On day 45, no treatment differences were observed.

**Table 3.** Effects of hydrolyzed yeast (HY) and carbadox on serum cytokine profile (fluorescence intensity values) and stress-relevant blood antioxidant criteria (SOD and TAC)^1^^,^^2^^,^^3^

								*P* ^5^			
Carbadox^4^:	Yes		No					HY in No Carbadox^6^			
HY, %:	0.00	0.04	0.00	0.04	0.08	0.12	SEM	Linear	Quadratic	0.04% HY^7^	Carbadox^8^
GM-CSF^9^											
day 10	32.2	17.2	20.4	17.4	17.6	105.4	1.40	0.001	0.003	0.231	0.491
day 45	9.5	9.1	8.1	8.4	9.8	11.4	1.40	0.428	0.846	0.981	0.707
IFNγ											
day 10^10^	229.5	107.2	103.6	98.1	106.0	330.0	1.39	0.015	0.065	0.205	0.170
day 45	21.0	20.5	19.9	29.0	27.9	36.2	1.39	0.226	0.862	0.582	0.647
IL-1α											
day 10^10^	262.4	87.8	241.3	196.1	88.7	321.7	1.51	0.970	0.064	0.107	0.371
day 45	72.3	44.5	57.5	75.1	43.3	91.8	1.51	0.637	0.546	0.786	0.714
IL-1β											
day 10	262.7	113.6	236.4	193.8	105.2	338.3	1.47	0.784	0.071	0.170	0.570
day 45	62.5	35.6	36.8	45.2	28.9	59.2	1.47	0.563	0.496	0.636	0.697
IL-1ra											
day 10	541.3	331.2	480.1	401.7	348.2	629.4	1.32	0.586	0.159	0.220	0.894
day 45	83.0	66.8	76.3	81.5	60.6	113.6	1.32	0.466	0.303	0.783	0.833
IL-2											
day 10^10^	246.7	82.4	233.1	168.2	72.3	292.2	1.56	0.932	0.049	0.103	0.450
day 45	50.9	32.8	41.6	57.3	32.7	66.5	1.56	0.666	0.651	0.888	0.681
IL-4											
day 10^10^	268.6	102.3	249.0	191.3	90.3	356.7	1.51	0.855	0.041	0.123	0.488
day 45	54.3	39.4	42.8	65.6	42.9	77.9	1.51	0.443	0.831	0.893	0.732
IL-6											
day 10^10^	255.5	98.2	244.7	177.4	94.8	313.8	1.49	0.946	0.053	0.102	0.481
day 45	50.2	29.7	40.8	43.4	27.8	59.5	1.49	0.698	0.369	0.551	0.823
IL-8											
day 10	767.8	430.0	867.2	564.1	797.9	698.6	1.34	0.812	0.597	0.074	0.485
day 45	138.5	91.5	97.4	117.2	146.8	96.9	1.34	0.869	0.287	0.683	0.854
IL-10											
day 10^10^	267.4	98.7	259.8	182.4	95.2	303.6	1.52	0.921	0.063	0.097	0.471
day 45	44.5	27.1	33.7	41.0	29.3	49.5	1.52	0.655	0.686	0.711	0.864

IL-12											
day 10	777.8	801.1	838.7	673.4	836.0	878.3	1.32	0.768	0.615	0.722	0.854
day 45	333.4	294.0	243.1	220.2	251.0	305.2	1.32	0.500	0.581	0.674	0.258
IL-18											
day 10^10^	312.1	137.8	265.9	242.6	158.2	406.4	1.40	0.570	0.118	0.169	0.539
day 45	75.5	48.5	49.7	66.1	52.4	74.8	1.40	0.504	0.915	0.811	0.869
TNFα											
day 10	89.7	50.9	42.0	52.5	41.9	174.9	1.32	0.001	0.025	0.523	0.175
day 45	26.1	21.7	18.7	20.3	19.1	28.5	1.32	0.324	0.548	0.848	0.459
TAC, mMol CRE											
day 10	585.5	594.1	618.2	625.6	605.3	611.1	32.24	0.773	0.980	0.805	0.322
day 45	729.6	750.0	744.1	748.9	680.4	637.0	32.24	0.008	0.456	0.695	0.836
SOD, U/mL											
day 10^10^	2.57	3.49	3.31	3.15	2.53	2.56	0.376	0.095	0.808	0.312	0.595
day 45	3.72	4.09	3.85	3.61	3.47	3.58	0.241	0.376	0.470	0.796	0.470

1 A total of 360 pigs (initially 5.4 ± 0.01 kg) were used in a 45-d growth study with 5 pigs per pen and 12 replicates per treatment (*N* = 12). Serum cytokine profile was evaluated at Eve Technologies (Calgary, AB, Canada). Data were log10 transformed for statistical analysis and transformed back for the cell mean values reported in this table.

2 No treatment × day interactions (*P *> 0.05).

3 SOD; superoxide dismutase. TAC; total antioxidant capacity.

4 Mecadox 2.5 (Phibro; Teaneck, NJ) included at 55 mg/kg of complete feed.

5 No interactive responses (*P *> 0.10) of carbadox and HY were observed.

6 Increasing HY in diets without carbadox.

7 Comparing the mean of 0% HY with and without carbadox vs. the mean of 0.04% HY with and without carbadox.

8 Comparing the mean of 0% and 0.04% HY with carbadox vs. the mean of 0% and 0.04% HY without carbadox.

9 Granulocyte-macrophage colony-stimulating factor.

10 Pairwise comparison of carbadox with 0.04% HY vs. carbadox without HY, *P *< 0.10.

### Antioxidant status

No treatment differences were observed on day 10 for TAC. Total antioxidant capacity decreased (linear, *P *= 0.008) on day 45 as HY increased. Increasing HY tended to decrease (linear, *P *= 0.095) serum SOD activity on day 10. Also, there was a tendency for an increase (*P *< 0.10) in serum SOD activity in pigs fed 0.04% HY with carbadox compared to pigs fed carbadox without HY. No treatment differences were observed on day 45.

## Discussion

*Saccharomyces cerevisiae* yeast products are available in various forms, including live yeast (probiotic), yeast cultures (prebiotic), yeast extracts (postbiotic), yeast cell walls, and hydrolyzed yeast. Research on yeast-based products primarily focus on nursery pigs, as they are highly susceptible to gut pathogens and diarrhea due to dietary transitions, immature gut health, and social stressors (Chance et al., 2021; Long et al., 2021). Studies have reported benefits of yeast supplementation on immune function and gut morphology (Shen et al., 2009; Namted et al., 2022). Additionally, yeast products contain nucleotides, free amino acids, and vitamins (Demirgul et al., 2022), which can support intestinal development and may contribute to improved nutrient digestibility. However, these positive effects do not always translate into improved growth performance, likely due to the variability in yeast composition due to differences in manufacturing processes, culture conditions, and preparation techniques (Aguilar-Uscanga & Francois, 2003).

Some studies have demonstrated positive effects of added yeast products on weanling pig growth performance. For instance, Boontiam et al. (2022) observed a linear increase in BW and ADG with increasing hydrolyzed yeast (0%, 5%, and 10% of the diet). These improvements can be attributed to enhanced crude protein digestibility, and improved intestinal morphology (Boontiam et al., 2022). Similarly, Shi and Kim (2019) reported improvements in growth performance with added yeast extract complex (0.1% and 0.2%) from a mixture of *Kluyveromyces maxianus* and *S. cerevisiae* containing β-glucan, mannan, nucleotide protease, and α-amylase, noting increased overall ADG and a tendency for increased ADFI, along with a tendency for increased G:F when pigs were approximately 25 kg. In contrast, the present study observed no differences in ADG or ADFI, despite containing similar components of β-glucan, mannan, and enzymes, potentially due to the considerably lower inclusion rates of HY (0.04%, 0.08%, and 0.12%). Molist et al. (2014) also did not observe differences in growth performance when supplementing 0.2% of a different hydrolyzed *S. cerevisiae* yeast derivative. However, they observed a tendency for increased G:F, which is comparable to the increased G:F in the present study with 0.04% added HY. Similarly, Liu et al. (2017) observed increased G:F when supplementing *S. cerevisiae* cell wall extract at 0.5% and 1.0% of the diet. These findings illustrate the potential of *S. cerevisiae*-derived products to improve G:F, although responses may vary depending on the specific form and inclusion level.

Carbadox is used in swine production due to its ability to regulate gut microbiota (Looft et al., 2014), promote growth and enhance G:F (Yen et al., 1985), as well as its capacity to reduce inflammation (He et al., 2020). By suppressing the proliferation of gram-negative bacteria in the small intestine such as ETEC, carbadox can improve digestion and nutrient absorption by reducing microbial competition, ultimately improving growth performance (Gaskins et al., 2002). Although *E coli* enumeration was not evaluated in the current study, the addition of carbadox increased fecal DM and decreased the frequency of diarrhea. Although HY is being explored as a potential alternative for carbadox, it does not have the same immediate antimicrobial potency. Yeast products work mainly through immunomodulation and prebiotic effects, indirectly changing the microbiota (Zanello et al., 2011).

Research on the effects of carbadox dating back to the late 1960s supports its ability to improve growth performance in nursery pigs (Thrasher et al., 1969; Roof and Mahan, 1982; Nabuurs et al., 1990). The findings from the present study demonstrate that added carbadox improved overall ADG, ADFI, and BW gain compared to pigs fed diets without carbadox. Notably, the increase in BW gain occurred without a proportional improvement in G:F, suggesting that the enhanced growth performance was primarily driven by greater feed intake. This observation aligns with Yen and Pond (1993), who observed that pigs fed carbadox at 55 mg/kg exhibited greater BW and a tendency for an increased feed intake without changes in G:F.

Oxidative stress in weanling pigs can result from various factors like environmental stressors, disease, and dysbiosis in the gut microbiome (Lykkesfeldt and Svendsen, 2007). Total antioxidant capacity measures the overall antioxidant potential in a biological sample, while SOD specifically quantifies the activity of an enzyme crucial to the antioxidant defense system (Bafana et al., 2011). While there are no established reference values for optimal TAC and SOD levels, higher concentrations generally indicate improved antioxidant status. Previous research has shown that including yeast-based products like cell wall extract at either 0.05%, 0.10%, or 0.15% (Liu et al., 2017) and live yeast at 0.2% (Long et al., 2021) in nursery pig diets can enhance SOD activity, suggesting an increase in antioxidative capacity. In contrast, the present study observed no differences in SOD activity or TAC levels between pigs fed the control diet and 0.04% added HY. However, an unexpected decrease in both TAC and SOD were observed with increasing HY, indicating a reduction in antioxidant capacity. A possible explanation is that higher inclusions of HY may have been potentially acting as a physiological stressor rather than providing a beneficial effect on oxidative status, as indicated by the elevated cytokines observed with this diet.

Cytokines are signaling proteins that mediate communication between immune cells and regulate inflammation, tissue repair, and immune responses. A decrease in pro-inflammatory cytokine levels such as interferon gamma (IFNγ), interleukins (IL-1β, IL-2, IL-6, IL-8, IL-12, and IL-18), and tumor necrosis factor alpha (TNFα) indicate improved inflammation status, suggesting that pigs may allocate less energy and AA toward immune overexpression, potentially enhancing overall energy and AA utilization (Dinarello, 2000). Anti-inflammatory cytokines such as IL-4 and IL-10 reduce inflammation and regulate the immune response by reducing the pro-inflammatory cytokine response. Christensen et al. (2022) observed no differences in cytokines IL-6, IL-10, or TNFα in pigs fed diets with enzymatically treated yeast compared with pigs fed the control diet. Fu et al. (2019) observed no differences in cytokines IL-1β or TNFα, but an increase in IL-10 in pigs fed a diet with yeast hydrolysates compared with those fed a control diet. Interleukins are a group of cytokines that act as chemical signals between white blood cells while IFN help resist viral infections and regulate innate and adaptive immune responses. Zanello et al. (2011) observed that yeast culture reduced the intestinal inflammation caused by pathogens through the inhibition of pro-inflammatory cytokine expression (TNFα, IL-1α, IL-6, and IL-8) induced by ETEC. In the present study, 0.04% HY inclusion with carbadox tended to decrease IFNγ, IL-1α, IL-2, IL-4, IL-6, IL-10, and IL-18 compared to feeding carbadox without HY supplementation, suggesting that these pigs had less inflammation or immune activation, which indicates an additive effect of the two products. Granulocyte-macrophage colony-stimulating factor (GM-CSF) is produced by activated T lymphocytes and other immune cells to control the differentiation and functional activation of hematopoietic cells following infection. Cytokines GM-CSF, IL-2, IL-4, and TNFα decreased then increased on day 10 and IL-1α, IL-1β, IL-6, and IL-10 tended to decrease then increase with increasing HY in diets without carbadox, again suggesting that higher inclusions of HY may have caused a physiological stressor as the cytokine levels were high. The observed increase in cytokines levels may be attributed to the yeast components, specifically β-glucans, which are recognized by receptors like dectin-1 on immune cells such as macrophages and neutrophils (Goodridge et al., 2009). The binding of β-glucans can trigger the production of pro-inflammatory cytokines such as TNF-α and IL-1β (Fu et al., 2023). In the present study, carbadox had no effect on cytokine concentrations. However, previous literature has shown that carbadox can reduce pro-inflammatory cytokines like TNFα (He et al., 2020) and IL-1β (Hung et al., 2020).

Complex carbohydrates, such as β-glucans and MOS, impact the gut microbiota of the pig by binding pathogenic bacteria like *E. coli* and Salmonella, decreasing their ability to adhere to intestinal walls (Moreira dos Anjos et al., 2019). These components are found in products like hydrolyzed yeast that contain components of the yeast cell wall. Lee et al. (2021) observed that an inclusion of 0.05% yeast cell wall tended to decrease the frequency of diarrhea compared to the control diet during the first 2 wk post-weaning. A similar finding was observed in the present study, where pigs fed diets containing HY had higher fecal DM (indicating lower diarrhea) than those fed the control diet. Due to their ability to bind pathogenic bacteria, yeast-products containing β-glucans and MOS have been widely studied for their effects in challenge models. Trckova et al. (2014) found that pigs fed live yeast *S. cerevisiae* at 0.5% had a lower daily diarrhea score, indicating less diarrhea, as well as a shorter duration of diarrhea compared to the control group when the pigs are orally challenged with ETEC K88. Although pigs were not inoculated with *E. coli* in the present study, *E. coli* can still be present under non-challenge conditions. Through diagnostic testing of a representative fecal sample for this group of pigs, F18 *E. coli* was identified via polymerase chain reaction analysis with genes for heat stable toxin 1a (st1a), heat stable toxin 1b (st1b), and heat labile toxin (lt) along with rotavirus group A. A similar response in a non-challenge setting was observed by Boontiam et al. (2020) who noted lower diarrhea rate with increasing hydrolyzed yeast of a different yeast product, aligning with the findings from the present study where there was an increase in fecal DM as HY inclusion increased.

Additionally, pigs fed diets containing carbadox had a higher fecal DM. Carbadox inhibits the growth of enteric pathogens and alters the gut microbial composition, which can lead to decreased prevenance of diarrhea. Because of this, more research has been done evaluating its effect in pathogen challenges. Kim et al. (2021) observed that ETEC F18 challenged pigs fed diets containing carbadox had lower average diarrhea scores and frequency of diarrhea, like observed in pigs fed carbadox in the present study. In the present study, there was a correlation between diarrhea occurrence, as measured by fecal scoring, and fecal DM. As HY inclusion increased, the frequency of diarrhea decreased and fecal DM increased, indicating firmer stools. Although increased stool consistency may indicate improved gut health, the effect may be insufficient to result in improved growth performance (Lee et al., 2021). Growth performance is multifactorial, being influenced by factors such as nutrient digestibility, while immune system activation can divert nutrients away from growth.

In conclusion, 0.04% HY improved G:F and increasing HY improved fecal DM in the early nursery phase. However, increasing HY inclusion did not result in improved growth performance. Pigs fed carbadox had increased final BW and firmer stools compared with those fed no carbadox and the same HY inclusion. Addition of 0.04% HY with carbadox tended to reduce serum cytokine concentrations (IFNγ, IL-1α, IL-2, IL-4, IL-6, IL-10, and IL-18) compared to carbadox alone. Additionally, serum cytokine concentrations (TNFα, IL-1α, IL-1β, IL-2, IL-4, IL-6, and IL-10) tended to decrease then increase as HY increased with the lowest concentration observed at an inclusion level of 0.08%. The combination of HY with carbadox showed potential immunomodulatory effects by reducing cytokine concentrations, suggesting HY may support immune regulation, but benefits on growth were limited.

## Abbreviations:

ADG: average daily gain
ADFI: average daily feed intake
AGP: antibiotic growth promoter
BW: body weight
DM: dry matter
ESBM: enzymatically treated soybean meal
ETEC: enterotoxigenic E. coli
G:F: gain:feed ratio
GM-CSF: granulocyte-macrophage colony-stimulating factor
HY: hydrolyzed yeast
IFN: interferon
IL: interleukin
MOS: mannan-oligosaccharides
NC: negative control
PC: positive control
SBM: soybean meal
SID: standardized ileal digestible
SOD: superoxide dismutase
TAC: total antioxidant capacity

## References

[skaf331-B1] Aguilar-Uscanga B. , Francois J. M. 2003. A study of the yeast cell wall composition and structure in response to growth conditions and mode of cultivation. Lett. Appl. Microbiol. 37:268–274. 10.1046/j.1472-765X.2003.01394.x 12904232

[skaf331-B2] Bafana A. , Dutt S., Kumar A., Kumar S., Ahuja P. S. 2011. The basic and applied aspects of superoxide dismutase. J. Mol. Catal. B. Enzym. 68:129–138. 10.1016/j.molcatb.2010.11.007

[skaf331-B3] Boontiam W. , Bunchasak C., Kim Y. Y., Kitipongpysan S., Hong J. 2022. Hydrolyzed yeast supplementation to newly weaned piglets: growth performance, gut health, and microbial fermentation. Animals 12:350. 10.3390/ani12030350 35158673 PMC8833445

[skaf331-B4] Boontiam W. , Wachirapakorn C., Phaengphairee P. 2020. Effects of hydrolyzed yeast supplementation on growth performance, immunity, antioxidant capacity, and microbial shedding in weaning pigs. Vet. World 13:1902–1909. 10.14202/vetworld.2020.1902-1909 33132604 PMC7566246

[skaf331-B5] Breen E. J. , Polaskova V., Khan A. 2015. Bead-based multiplex immuno-assays for cytokines, chemokines, growth factors and other analytes: median fluorescence intensities versus their derived absolute concentration values for statistical analysis. Cytokine 71:188–198. 10.1016/j.cyto.2014.10.030 25461398

[skaf331-B6] Campbell J. M. , Crenshaw J. D., Polo J. 2013. The biological stress of early weaned piglets. J. Anim. Sci. Biotechnol. 4:19. 10.1186/2049-1891-4-19 23631414 PMC3651348

[skaf331-B7] Chance J. A. , DeRouchey J. M., Amachawadi R. G., Ishengoma V., Nagaraja T. G., Goodband R. D., Woodworth J. C., Tokach M. D., Calderón H. I., Kang Q., Loughmiller J. A., Hotze B., Gebhardt J. T. 2021. Live yeast and yeast extracts with and without pharmacological levels of zinc on nursery pig growth performance and antimicrobial susceptibilities of fecal *Escherichia coli*. J. Anim. Sci. 99:1–10. 10.1093/jas/skab330 PMC8664753 34752618

[skaf331-B8] Chen Q. , Tang S., Jin X., Zou J., Chen K., Zhang T., Xiao X. 2009. Investigation of the genotoxicity of quinocetone, carbadox and olaquindox in vitro using Vero cells. Food Chem. Toxicol. 47:328–334. 10.1016/j.fct.2008.11.020 19061932

[skaf331-B9] Christensen B. , Zhu C., Mohammadigheisar M., Schulze H., Huber L.-A., Kiarie E. G. 2022. Growth performance, immune status, gastrointestinal tract ecology, and function in nursery pigs fed enzymatically treated yeast without or with pharmacological levels of zinc. J. Anim. Sci. 100:1–14. 10.1093/jas/skac094 PMC9047176 35323958

[skaf331-B10] Dasenaki M. E. , Kritikou A. S., Thomaidis N. S. 2023. Meat safety: II residues and contaminants. In: Toldrá F., editor. Lawrie’s Meat Science. Cambridge: Elsevier; p. 591–626. 10.1016/B978-0-323-85408-5.00007-8

[skaf331-B11] Demirgul F. , Simsek O., Sagdic O. 2022. Amino acid, mineral, vitamin B contents and bioactivities of extracts of yeasts isolated from sourdough. Food Biosci. 50:102040. 10.1016/j.fbio.2022.102040

[skaf331-B12] Dinarello C. A. 2000. Proinflammatory cytokines. CHEST. 118:503–508. 10.1378/chest.118.2.503 10936147

[skaf331-B13] Dritz S. S. , Tokach M. D., Goodband R. D., Nelssen J. L. 2002. Effects of administration of antimicrobials in feed on growth rate and feed efficiency of pigs in multisite production systems. J. Amer. Vet. Med. Assoc. 220:1690–1695. 10.2460/javma.2002.220.1690 12051512

[skaf331-B14] Fairbrother J. M. , Nadeau É., Gyles C. L. 2005. *Escherichia coli* in postweaning diarrhea in pigs: an update on bacterial types, pathogenesis, and prevention strategies. Anim. Health. Res. Rev. 6:17–39. 10.1079/AHR2005105 16164007

[skaf331-B15] FDA (Food and Drug Administration). 2013. New animal drugs and new animal drug combination products administered in or on medicated feed or drinking water of food-producing animals: recommendations for drug sponsors for voluntarily aligning product use conditions with GFI #209. FDA Guidance for Industry #213. https://www.fda.gov/ media/83488/download.

[skaf331-B16] Fu R. , Chen D., Tian G., Zheng P., Mao X., Yu J., He J., Huang Z., Luo Y., Yu B. 2019. Effect of dietary supplementation of *Bacillus coagulans* or yeast hydrolysates on growth performance, antioxidant activity, cytokines and intestinal microflora of growing-finishing pigs. Anim. Nutr. 5:366–372. 10.1016/j.aninu.2019.06.003 31890913 PMC6920390

[skaf331-B17] Fu R. , Liang C., Chen D., Tian G., Zheng P., He J., Yu J., Mao X., Luo Y., Luo J., Yu B. 2023. Yeast hydrolysate attenuates lipopolysaccharide-induced inflammatory responses and intestinal barrier damage in weaned piglet. J. Anim. Sci. Biotechnol. 14:44. 10.21203/rs.3.rs-2005990/v1 36932457 PMC10021991

[skaf331-B18] Gaskins H. R. , Collier C. T., Anderson D. B. 2002. Antibiotics as growth promotants: mode of action. Anim. Biotechnol. 13:29–42. 10.1081/ABIO-120005768 12212942

[skaf331-B19] Goodridge H. S. , Wolf A. J., Underhill D. M. 2009. β‐glucan recognition by the innate immune system. Immunol. Rev. 230:38–50. 10.1111/j.1600-065X.2009.00793.x 19594628 PMC6618291

[skaf331-B20] He Y. , Kim K., Kovanda L., Jinno C., Song M., Chase J., Li X., Tan B., Liu Y. 2020. Bacillus subtilis: a potential growth promoter in weaned pigs in comparison to carbadox. J. Anim. Sci. 98:1–13. 10.1093/jas/skaa290 PMC7523599 32877510

[skaf331-B21] Hung Y.-T. , Hu Q., Faris R. J., Guo J., Urriola P. E., Shurson G. C., Chen C., Saqui-Salces M. 2020. Analysis of gastrointestinal responses revealed both shared and specific targets of zinc oxide and carbadox in weaned pigs. Antibiotics 9:463. 10.3390/antibiotics9080463 32751572 PMC7460413

[skaf331-B22] Kim K. , He Y., Jinno C., Kovanda L., Li X., Song M., Liu Y. 2021. Trace amounts of antibiotic exacerbated diarrhea and systemic inflammation of weaned pigs infected with a pathogenic *Escherichia coli*. J. Anim. Sci. 99:1–13. 10.1093/jas/skab073 PMC8480179 33693730

[skaf331-B23] Kluess J. , Schoenhusen U., Souffrant W. B., Jones P. H., Miller B. G. 2010. Impact of diet composition on ileal digestibility and small intestinal morphology in early-weaned pigs fitted with a T-cannula. Animal 4:586–594. 10.1017/S1751731109991455 22444046

[skaf331-B24] Lee J. J. , Kyoung H., Cho J. H., Choe J., Kim Y., Liu Y., Kang J., Lee H., Kim H. B., Song M. 2021. Dietary yeast cell wall improves growth performance and prevents diarrhea of weaned pigs by enhancing gut health and anti-inflammatory immune responses. Animals. 11:2269. 10.3390/ani11082269 34438727 PMC8388398

[skaf331-B25] Liu G. , Yu L., Martínez Y., Ren W., Ni H., Abdullah Al-Dhabi N., Duraipandiyan V., Yin Y. 2017. Dietary *Saccharomyces cerevisiae* cell wall extract supplementation alleviates oxidative stress and modulates serum amino acids profiles in weaned piglets. Oxid. Med. Cell. Longev. 2017:3967439. 10.1155/2017/3967439 28386308 PMC5366236

[skaf331-B26] Long S. , He T., Kim S. W., Shang Q., Kiros T., Mahfuz S. U., Wang C., Piao X. 2021. Live yeast or live yeast combined with zinc oxide enhanced growth performance, antioxidative capacity, immunoglobulins and gut health in nursery pigs. Animals. 11:1626. 10.3390/ani11061626 34072877 PMC8228624

[skaf331-B27] Looft T. , Allen H. K., Casey T. A., Alt D. P., Stanton T. B. 2014. Carbadox has both temporary and lasting effects on the swine gut microbiota. Front. Microbiol. 5:276. 10.3389/fmicb.2014.00276 24959163 PMC4050737

[skaf331-B28] Lykkesfeldt J. , Svendsen O. 2007. Oxidants and antioxidants in disease: Oxidative stress in farm animals. Vet. J. 173:502–511. 10.1016/j.tvjl.2006.06.005 16914330

[skaf331-B29] Molist F. , Van Eerden E., Parmentier H. K., Vuorenmaa J. 2014. Effects of inclusion of hydrolyzed yeast on the immune response and performance of piglets after weaning. Anim. Feed Sci. Technol. 195:136–141. 10.1016/j.anifeedsci.2014.04.020

[skaf331-B30] Moreira dos Anjos C. , Dias Gois F., Moreira dos Anjos C., de Souza Rocha V., de Sá e Castro D.E., Bezerra Allaman I., Silva F. Lessa, de Oliveira Carvalho P.L., Meneghetti C., Costa L.B. 2019. Effects of dietary beta-glucans, glucomannans and mannan oligosaccharides or chlorohydroxyquinoline on the performance, diarrhea, hematological parameters, organ weight and intestinal health of weanling pigs. Livest. Sci. 223:39–46. 10.1016/j.livsci.2019.02.018

[skaf331-B31] Nabuurs M. J. A. , Van Der Molen E. J., De Graaf G. J., Jager L. P. 1990. Clinical signs and performance of pigs treated with different doses of carbadox, cyadox and olaquindox. J. Vet. Med. 37:68–76. 10.1111/j.1439-0442.1990.tb00877.x 2110404

[skaf331-B32] Namted S. , Poungpong K., Loongyai W., Rakangthong C., Bunchasak C. 2022. Dietary autolysed yeast modulates blood profiles, small intestinal morphology and caecal microbiota of weaning pigs. Animal 16:100660. 10.1016/j.animal.2022.100660 36279713

[skaf331-B33] NRC. 2012. Nutrient requirements of swine, 11th ed. Washington D.C: Natl. Acad. Press.

[skaf331-B34] Roof M. D. , Mahan D. C. 1982. Effect of carbadox and various dietary copper levels for weanling swine. J. Anim. Sci. 55:1109–1117. 10.2527/jas1982.5551109x 7174553

[skaf331-B35] Sampath V. , Sureshkumar S., Kim I. H. 2023. The efficacy of yeast supplementation on monogastric animal performance. Life 13:2037. 10.3390/life13102037 37895419 PMC10608604

[skaf331-B36] Schiavone M. , Sieczkowski N., Castex M., Dague E., Marie François J. 2015. Effects of the strain background and autolysis process on the composition and biophysical properties of the cell wall from two different industrial yeasts. FEMS Yeast Res. 15:1–11. 10.1093/femsyr/fou012 25762053

[skaf331-B37] Shen Y. B. , Piao X. S., Kim S. W., Wang L., Liu P., Yoon I., Zhen Y. G. 2009. Effects of yeast culture supplementation on growth performance, intestinal health, and immune response of nursery pigs. J. Anim. Sci. 87:2614–2624. 10.2527/jas.2008-1512 19395514

[skaf331-B38] Shi H. , Kim I. H. 2019. Dietary yeast extract complex supplementation increases growth performance and nutrient digestibility of weaning pigs. Livest. Sci. 230:103850. 10.1016/j.livsci.2019.103850

[skaf331-B39] Thrasher G. W. , Shively J. E., Askelson C. E., Babcock W. E., Chalquest R. R. 1969. Effects of feeding carbadox upon the growth and performance of young pigs. J. Anim. Sci. 28:208–215. 10.2527/jas1969.282208x 5773530

[skaf331-B40] Trckova M. , Faldyna M., Alexa P., Zajacova Z. S., Gopfert E., Kumprechtova D., Auclair E., D’Inca R. 2014. The effects of live yeast Saccharomyces cerevisiae on postweaning diarrhea, immune response, and growth performance in weaned piglets. J. Anim. Sci. 92:767–774. 10.2527/jas.2013-6793 24305872

[skaf331-B41] Van Der Peet-Schwering C. M. C. , Jansman A. J. M., Smidt H., Yoon I. 2007. Effects of yeast culture on performance, gut integrity, and blood cell composition of weanling pigs. J. Anim. Sci. 85:3099–3109. 10.2527/jas.2007-0110 17609465

[skaf331-B42] Van Heugten E. , Funderburke D. W., Dorton K. L. 2003. Growth performance, nutrient digestibility, and fecal microflora in weanling pigs fed live yeast. J. Anim. Sci. 81:1004–1012. 10.2527/2003.8141004x 12723090

[skaf331-B43] Yan H. , Xing Q., Xiao X., Yu B., He J., Mao X., Yu J., Zheng P., Luo Y., Wu A., Pu J., Lu P., Wei M., Khafipour E., Chen D. 2024. Effect of *Saccharomyces cerevisiae* postbiotics and essential oil on growth performance and intestinal health of weanling pigs during K88 ETEC infection. J. Anim. Sci. 102:1–16. 10.1093/jas/skae007 PMC11087729 38198728

[skaf331-B44] Yen J. T. , Nienaber J. A., Pond W. G., Varel V. H. 1985. Effect of carbadox on growth, fasting metabolism, thyroid function and gastrointestinal tract in young pigs. J. Nutr. 115:970–979. 10.1093/jn/115.8.970 3926967

[skaf331-B45] Yen J. T. , Pond W. G. 1993. Effects of carbadox, copper, or *Yucca shidigera* extract on growth performance and visceral weight of young pigs. J. Anim. Sci. 71:2140–2146. 10.2527/1993.7182140x 8376238

[skaf331-B46] Zanello G. , Meurens F., Berri M., Chevaleyre C., Melo S., Auclair E., Salmon H. 2011. Saccharomyces cerevisiae decreases inflammatory responses induced by F4+ enterotoxigenic Escherichia coli in porcine intestinal epithelial cells. Vet. Immunol. Immunopathol. 141:133–138. 10.1016/j.vetimm.2011.01.018 21354630

